# Editorial: Leptospirosis: pathogenesis, clinical and epidemiological aspects

**DOI:** 10.3389/fcimb.2023.1210178

**Published:** 2023-05-09

**Authors:** Angela S. Barbosa, Kanitha Patarakul, Lourdes Isaac

**Affiliations:** ^1^ Laboratory of Bacteriology, Butantan Institute, São Paulo, Brazil; ^2^ Department of Microbiology, Faculty of Medicine, Chulalongkorn University, Bangkok, Thailand; ^3^ Chula Vaccine Research Center (Chula VRC), Center of Excellence in Vaccine Research and Development, Chulalongkorn University, Bangkok, Thailand; ^4^ Department of Immunology, Institute of Biomedical Sciences, University of São Paulo, São Paulo, Brazil

**Keywords:** leptospirosis, *Leptospira*, pathogenesis, immune response, vaccines

Leptospirosis is an infectious disease of worldwide distribution caused by pathogenic species of the genus *Leptospira*. The disease affects domestic and wild animals as well as humans, and each year approximately one million new cases of leptospirosis are notified globally, among which 5-10% progress to death (Costa et al., 2015). Developing a deeper understanding of the general mechanisms of *Leptospira* pathogenesis will certainly lead to advances on new diagnostic tools, prevention and treatment of this infectious disease.

This compilation of research and review articles provides updates on bacterial pathogenesis and immune responses, and approaches aspects related to vaccine development against leptospirosis, including new candidates and adjuvant formulations. Those studies may shed light on *Leptospira* pathogenesis at the molecular and physiological level, and impact on the treatment of this neglected infectious disease.

A short-term murine model that allows hematogenous dissemination of both pathogenic and non-pathogenic *Leptospira* was developed by Surdel et al. C3H/HeJ mice were inoculated intravenously, and tissues were harvested 24 hours post-infection. Interestingly, non-pathogenic *L. biflexa* serovar Patoc were isolated from multiple organs by qPCR, but showed lower burdens compared to the pathogenic *L. interrogans* serovar Manilae strain. Furthermore, non-pathogenic leptospires were detected for at least six hours post infection in the blood. This model can be useful to uncover novel virulence determinants involved in host colonization and immune evasion, having the benefit of allowing the use of both loss- and gain-of-function bacterial strains.

Taking advantage of this infection model, Surdel et al. assessed the *in vivo* role of the candidate adhesins LIC11574 and LIC13411, previously shown to bind to VE-cadherin *in vitro* (Evangelista et al., 2014a; Evangelista et al., 2014b). Heterologous production of LIC13411 in non-pathogenic *L. biflexa* increased binding of this saprophytic strain to human endothelial cells and specifically to VE-cadherin. In the short-term murine model of infection this gain-of-function strain showed increased burdens in multiple organs. Thus, tharapies targeting *Leptospira* adhesins may be an efficient way to prevent or limit infection by this spirochete.

The importance for searching new vaccine candidates against leptospirosis and the limits of using hamsters as an experimental model were highlighted in the study by Maia et al. The authors used 22 vaccine candidates selected by reverse vaccinology and immunized a relatively large number of hamsters before challenge with pathogenic *L. interrogans* Fiocruz L1-130. In two large experiments, the immunization with the recombinant proteins LIC11570, LIC13229, LIC13417 and LIC20214 conferred 100% protection, sterilizing immunity, and significant specific IgG production. However, these promising results were not reproduced in the third experiment. The authors suggest that future studies employ C3H/HeJ mice, since these animals are quite susceptible to leptospirosis: they are Toll-like receptor-4 deficient and therefore would not respond to the possible presence of *E. coli* LPS in the recombinant protein preparations. In addition, there is a larger repertoire of commercial reagents that can be used to characterize the mouse immune response against *Leptospira* during vaccination.

Novel, promising vaccine candidates against leptospirosis were reported by Chaurasia et al.: the PF07598 Gene Family-Encoded Virulence Modifying Proteins (VM proteins). Those secreted toxins were previously shown to promote cytotoxicity *in vitro* (Chaurasia et al., 2022). C3H/HeJ mice immunized with recombinant VM proteins and challenged with *L.interrogans* serovar Canicola were protected and had a significant reduction in bacterial load in the liver and kidney. These data pave the way for the development of a pan-leptospirosis subunit vaccine.

Adjuvants are being studied to enhance the efficacy of subunit vaccines to provide complete protection against leptospirosis. The LMQ (neutral liposomes containing monophosphoryl lipid A (MPL) and *Quillaja saponaria* derived QS21 saponin) adjuvant has previously been shown to be an effective adjuvant for the C-terminal region (domain 7-13) of leptospiral immunoglobulin-like protein A (LigAc) (Techawiwattanaboon et al., 2020), one of the most promising vaccine candidates. The study by Techawiwattanaboon et al. evaluated the effectiveness of three alternative adjuvants (LQ, LQuil, and SQuil) in promoting immunogenicity and protective efficacy. Results indicate that these adjuvants confer substantial antibody responses and protective efficacy comparable to the LMQ adjuvant, indicating their potential for improving human and animal leptospirosis vaccines.

The role of secreted proteins in the pathogenesis of *Leptospira* is still an unexplored area. Courrol et al. characterized *L. interrogans* leptolysin, a secreted metalloprotease that targets proteinaceous substrates such as proteoglycans and plasma fibronectin. Leptolysin is highly conserved among *Leptospira* species belonging to the subclade P1. Its broad-spectrum proteolytic activity substantiates future *in vivo* studies that may provide further evidence of the involvement of this protease in the pathogenesis of leptospirosis through inactivation of host proteins.

Leptospires, known as extracellular bacteria, have been a subject of controversy regarding their ability to replicate within macrophages. Santecchia et al. demonstrated that murine macrophages can internalize pathogenic leptospires *in vivo*. Human (PMA-differentiated) THP1 and mouse RAW264.7 macrophage cell lines were actively infected by three different serotypes of pathogenic *L. interrogans* and the saprophytic *L. biflexa*. However, the observed reduction in the intracellular load was not attributed to classical macrophage microbicidal mechanisms, but rather to an active exit of the bacteria. These findings indicate that leptospires do not exhibit an intracellular lifestyle in macrophages.

The mechanisms underlying mammalian susceptibility to leptospirosis remain poorly understood. Bonhomme et al. review recent research that suggests leptospires can avoid recognition by certain Toll-like and NOD-like receptors, including TLR4, TLR5, and NOD1, in various hosts, while TLR2 and NLRP3 responses are consistently observed across hosts. It is proposed that the innate immune mechanisms, particularly TLR4, could potentially determine susceptibility to leptospirosis in various hosts.

## Author contributions

All authors listed have made a substantial, direct, and intellectual contribution to the work and approved it for publication.
